# Performance Analysis of the IEEE 802.15.4 Protocol for Smart Environments under Jamming Attacks

**DOI:** 10.3390/s21124079

**Published:** 2021-06-13

**Authors:** Nicolás López-Vilos, Claudio Valencia-Cordero, Cesar Azurdia-Meza, Samuel Montejo-Sánchez, Samuel Baraldi Mafra

**Affiliations:** 1 Department of Electrical Engineering, Universidad de Chile, Santiago 8370451, Chile; nicolas.lopez.v@usach.cl (N.L.-V.); cazurdia@ing.uchile.cl (C.A.-M.); 2 Department of Electrical Engineering, Universidad de Santiago de Chile, Santiago 9170124, Chile; 3 Programa Institucional de Fomento a la I+D+i, Universidad Tecnológica Metropolitana, Santiago 8940577, Chile; smontejo@utem.cl; 4 National Institute of Telecommunications (Inatel), Santa Rita do Sapucaí, MG 37540-000, Brazil; samuelbmafra@inatel.br

**Keywords:** cybersecurity, IEEE 802.15.4, Internet of Things (IoT), privacy, security, wireless sensor networks

## Abstract

Jamming attacks in wireless sensor networks (WSNs) scenarios are detrimental to the performance of these networks and affect the security and stability of the service perceived by users. Therefore, the evaluation of the effectiveness of smart environment platforms based on WSNs has to consider the system performance when data collection is executed under jamming attacks. In this work, we propose an experimental testbed to analyze the performance of a WSN using the IEEE 802.15.4 CSMA/CA unslotted mode under jamming attacks in terms of goodput, packet receive rate (PRR), and energy consumption to assess the risk for users and the network in the smart scenario. The experimental results show that constant and reactive jamming strategies severely impact the evaluated performance metrics and the variance’ of the received signal strength (RSS) for some signal-to-interference-plus-noise ratio (SINR) ranges. The measurements obtained using the experimental testbed were correlated with analytical models. The results show that in the presence of one interferer, for SINR values higher than 4.5 dB, the PRR is almost 0.99, and the goodput 3.05 Kbps, but the system performance is significantly degraded when the amount of interferers increases. Additionally, the energy efficiency associated with reactive strategies is superior to the constant attack strategy. Finally, based on the evaluated metrics and with the proposed experimental testbed, our findings offer a better understanding of jamming attacks on the sensor devices in real smart scenarios.

## 1. Introduction

Currently, the integration of the Internet of Things (IoT) to acquire data from several environments has shown a rapid expansion in various areas such as the industrial, farming, health, and smart cities [1]. This integration of the IoT in different contexts, contributes to deploy “smart” devices that expand the functionalities of the communication and control systems in these areas, generating smart environments. However, the notable benefits of the IoT with the integration of “smart devices” have also brought several challenges in terms of reliability, privacy, and security requirements [2].

The smart devices used in the deployment of the IoT in different environments employ the wireless medium to exchange the data across the network and to the cloud. As a consequence, the transmitted data and the integrity of the network is exposed to several cyberattacks such as tampering, eavesdropping, Denial of Service (DoS), and jamming attacks that diminish the reliability, privacy, and security of the network [3]. These threats are the principal detected strategies that an attacker could adopt to compromise the cybersecurity in the perception layer of the IoT system.

The generation of interference that compromises the integrity and the security of the networks in the perception layer is known as jamming attacks [4]. These cyber-attacks are a type of Denial of Service (DoS) that diminishes the Quality of Service (QoS) parameters, such as the link quality of the channel, the reception and delay of the packets, and the energy consumption. The attacks are performed by a device known as a jammer, which emits radio signals that interfere with legitimate wireless users. There are various types of jamming attacks, such as the constant jammer, intermittent jammer, reactive jammer, and intelligent jammer [5]. Consequently, the presence of jamming attacks severely impact the performance and the security of the IoT networks and users, despite the communication technology used.

To overcome the jamming attacks, the performance metrics of Packet Data Rate (PDR), Received Signal Strength (RSS), and energy consumption are used to determine their presence in the research community [6]. The analysis of these metrics allows the generation of countermeasures and the adaptation of the mathematical models under this assumption. Consequently, experimental data of these performance metrics must be acquired to deal with the attacker in the different smart environments that integrate the IoT systems such as the Wireless Sensor Networks (WSN) technology.

### Related Work

Wireless networks are exposed to different strategies of cyberattacks that aim to diminish the privacy, reliability, and security of communications. Therefore, the users are in danger owing to the presence of attackers. Specifically, the jamming attacks are being studied in diverse scenarios to generate countermeasures [7]. In Industrial IoT systems (IIoT), the DoS attacks against cyber-physical production systems (CPPS) are particularly detrimental to the production and to the safety of the workers [8]. Therefore, the attention of these types of attacks are being increased owing to the notorious impact on the business and factories. In the case of smart environments such as farming, cities, healthcare, and home automation, the jamming attacks also present a serious issue. In the smart farming context, the presence of attackers creates an unsafe and unproductive environment [9]. Therefore, the economy and wellness of the persons in a nation could be undermined by the destruction of fields, flood farmlands, and over spray pesticides. As a consequence, governments have made efforts to protect the agricultural sector against terrorist attacks, known as agroterrorism [10].

In the urban context, the massive use of IoT solutions allows the development of smart cities. The IoT systems acquire several data such as the temperature of a house, the air quality on the streets, and monitoring of patients in an e-health context. Moreover, the number of IoT devices is expected to grow between 25 to 30 billion by 2022, with a contribution of 5.1 billion from the automotive sector. However, this expansion of IoT devices also incremented the number of cyberattacks that the devices are exposed [11]. Consequently, the privacy and security of the IoT systems have presented several threats and attacks that impact the quality of life and integrity of citizens in smart cities.

The Healthcare Internet-of-Things (H-IoT) integrates the use of IoT architecture with the health management of patients [12]. The H-IoT devices combine sensors and actuators that improve the diagnostic and treatments of patients. Some examples are smart continuous glucose monitors, connected inhalers, activity/heart-rate trackers, and smartwatches. Therefore, the presence of attacks in the physical layer leaves vulnerable the health of patients and requires the enhancement of the security in the communication system [13].

Despite the importance of the improvement and enhancement of security against cybersecurity attacks in the stated IoT environments, few works analyze and implement in real scenarios the presence of attackers in the physical layer using the standard IEEE 802.15.4 [14,15,16,17,18]. In these works, the analysis of different jamming attacks in a WSN is analyzed through different performance metrics. The most analyzed metric corresponds to the power of signals as Received Signal Strength (RSS), and the packets correctly received as Packet Reception Rate (PRR) or Packet Delivery Ratio (PDR). These metrics are analyzed comparing scenarios where the network in a star topology works in the presence and the absence of the jamming attacks.

The reactive jamming strategy analyzed in [15,16,17] shows that the attacker is hard to detect, but the RSS metric provides good insights to determine the presence of reactive jamming in the network [16]. Meanwhile, for other jamming strategies, such as constant jamming, the detection is easier using the RSS, PRR, or PDR metric [14,17]. Therefore, the PDR metrics are used to offer guidelines and generate countermeasures against the reactive jamming strategy [14,18]. Therefore, in our work, we choose the RSS, PRR, and energy consumption as the performance metrics to be analyzed in the different scenarios with the presence of reactive and constant jamming attacks.

However, to the best of the author’s knowledge, the correlation and extension of a probabilistic unslotted CSMA/CA MAC model using the standard IEEE 802.15.4 in the presence of jamming attacks have not been proposed. To overcome the gap in the literature, we implement an IEEE 802.15.4 CSMA/CA-based network. We also implement a sniffer in a Software Defined Radio (SDR) to analyze the behavior of the network, under jamming attacks, without intervention. Additionally, using the XBee devices and a Voltage Controlled Oscillator, we implement reactive and constant jamming strategies to analyze their impacts on performance metrics. Our main contribution is to present a critical study based on a successful experimental testbed that includes the impact of jamming attacks on the transceiver devices used in real smart scenarios. Consequently, we extend the experimental analysis to the probabilistic model to include reactive and constant jamming attacks under the assumptions used in our work. The main contributions of this paper are as follows:

- We correlate and extend the probabilistic unslotted CSMA/CA MAC model [19] for scenarios in the presence of jamming attacks;
- We propose a novel experimental testbed for the analysis of jamming attacks in an IEEE 802.15.4 network using CSMA/CA unslotted mode;
- We detail the impact of reactive and constant jamming strategies based on performance metrics such as PDR, goodput, energy consumption, and RSS.

Additionally, to the stated contributions, the analysis of the performance metrics acquired under the assumptions of the work gives valuable insights. Specifically, when a different number of jammers are used, the performance metrics have a distinctive pattern. Therefore, the analysis of these patterns provides guidelines to the generation of countermeasures against attackers in the context of smart environments. These analysis and pattern particularities are also seen and analyzed for the different jamming strategies, whose study and understanding can contribute to improve the detection systems.

The remainder of the paper is structured as follows: Section 2 provides a detailed literature review of the WSNs and the IEEE 802.15.4 standard with the CSMA/CA algorithm, and the jamming attacks. Section 3 provides the details of the proposed experimental testbed. The results and analysis of the experimental data in the different scenarios are detailed in Section 4. Finally, the conclusions are discussed in Section 5.

## 2. WSNs

The WSN is a type of wireless network that interconnects several devices to collect and process information autonomously and flexibly in various environments. Their well-known properties, such as as low cost, reliability, and simplicity have allowed massive implementation of WSN in smart environments, which help make human being’s lives better [20]. However, several cyber-attacks could be performed by attackers in the different layers that compose a WSN, which compromises the health and wellness of the persons.

The layers that compose a WSN will depend on the standard used by the network to exchange the data across the network. The WSN protocol stack can be divided into six parts: Physical Layer (PHY), Data Link Layer, Network Layer, Transmission Control Layer, Application Support Layer, and Network Management Layer. The standard IEEE 802.15.4 defines the PHY and MAC layer specifications, and the standards as ZigBee defines the uppers layers. Each of these layers has its intrinsic functions, tasks, and at the same time, vulnerabilities that an attacker can exploit.

In the jamming attacks, the physical layer is exposed to interference signals produced by an attacker that aims to compromise the radio frequency used by the sensor nodes in the WSN to transmit the data. The attacker uses a jammer device to compromise the data integrity and the communication process generating dummy packets or noise. The jammer device could be a legitimate node in the network captured by the attacker or an external device from the network. Consequently, to study the impact of the jammer in the network is mandatory to understand the standard definitions and behavior of the layers implemented in the WSN.

### 2.1. IEEE 802.15.4

The standard IEEE 802.15.4 [21] is the most used in the implementation of Wireless Sensor Networks (WSN). The WSN is a fundamental piece in the rapid growth of the Internet of Things (IoT) in many environments to acquire and transmit data.

**Figure 1:**
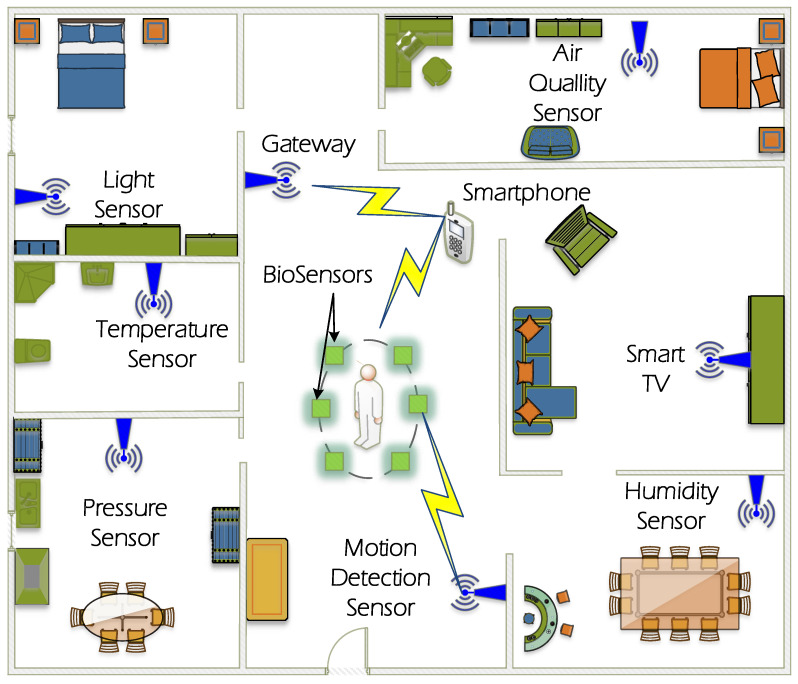
IoT devices are implemented in e-health environments for different monitoring purposes. Therefore, the existence of attackers that disrupt the 2.4 GHz ISM Band expose several risks to IoT devices.

In the smart context, the standard IEEE 802.15.4 is being used to transmit the data between smart devices as smartphones, smart TV, smart watch, health monitoring devices, and moreover as represented in Figure 1. Moreover, it is the base of other popular technologies such as ZigBee, 6LoWPAN, and WirelessHART that also aims to provide low power consumption of the devices, low rate transmission, and low cost of implementation.

The composition of an IEEE 802.15.4 network could be using different devices with singular functions and capabilities. The devices are divided into two classes: Full-Function Device (FFD) and Reducted-Function Device. The FFD devices communicate between devices of their similar or different class; on the contrary, the RFD devices only communicate with a similar class. As a consequence, only the FFD devices could assume the coordinator function in a network, and always at least one of these types of the device needs to be present in the conformation of a network. However, exist FFD that are programmed with the function of an end device as RFD in some applications.

Some application scenarios require different topologies of network to fulfill the requirements of the designer. The star and point-to-point (P2P) topologies are the defined arrangement of devices defined in the standard. In a P2P topology, devices only communicate with other devices in the range of communications. On the contrary, star topology admits communications between the coordinator and the other devices that are not necessarily in the transmission range of the coordinator. A representation of different devices that uses the IEEE 802.15.4 communication protocols in a Smart House are presented in Figure 1 to show the integration of the WSN devices in different rooms.

The PHY layer of IEEE 802.15.4 standard specifies the use of the 868/915 MHz and 2.4 GHz frequency bands to perform communications. The achievable data rates are 20 Kbps, 40 Kbps, and 250 Kbps, respectively. However, the 2.4 GHz ISM frequency band is used in smart context due to the requirements of the data rate that the applications demand. In the ISM band, O-QPSK modulation is used within the half-sine pulse for the pulse shaping process. Additionally, Direct Sequence Spread Spectrum (DSSS) transmit scheme is used to promote coexistence in the frequency band.

For the MAC layer of the IEEE 802.15.4 standard exist different options to fulfill the requirements in many applications. The algorithms Time Division Multiple Access (TDMA), Time Slotted Channel Hopping (TSCH), Carrier Sense Multiple Access (CSMA) are the possible configurations for the MAC layer. Specifically, the CSMA algorithm can be configured in two operations modes: slotted mode and unslotted mode. Additionally, it can be used in combination with Clear Channel Assessment (CCA) process to produce the Collision Avoidance improvement (CSMA/CA).

#### Unslotted CSMA/CA Mode

A device with a packet ready to be transmitted and uses the unslotted CSMA/CA mode with CCA will perform the following process: back off for a random integer value between $\left[0,2^{macMinBE}-1\right]$, before sensing the channel. The macMinBE corresponds to the minimum value of the Backoff Exponent (BE) configured in the device. Next, the device performs the CCA process to sense for activity in the channel and decide if it is assed busy or free. If the channel is detected busy, the BE is incremented by one, and a new backoff stage and sensing process starts. Then, the process will be repeated for the busy case until BE equals the macMaxBE parameter, which corresponds to the maximum number of BE. Additionally, if the algorithm reaches the maximum number of retransmissions macMaxFrameRetries, the packet will be discarded. However, if retransmissions are left in the process, the BE will be freeze at macMaxBE.

The used devices in this work use the following parameters to control the CSMA/CA unslotted mode with CCA. First, the device permits the control of macMinBe by the Random Delay Slots (RN) parameter, and the macMaxBe is always five by default. Next, the XBee Retries (RR) set the additional maximum number of retries that the device executes, but in this case, we set RR=0 to use the default values of retransmissions defined in the IEEE 802.15.4 standard. Finally, the CCA Threshold (CA) parameter sets the reference value of energy in the channel to report the state as free or busy. If the device detects energy above the CA parameter, it will not transmit the packet.

### 2.2. Analytical Model

The analytical model proposed by Buratti considers multiple nodes that transmit a packet to a single sink node [19,22]. The transmitting nodes accessing the channel can be in one of four states: backoff, sensing, transmission, or idle. Therefore, these node states are modeled as a bidimensional process $Q\left(t\right)=\{BO_{c}\left(t\right),BO_{s}\left(t\right)\}$ where t represents the time slot, which later is linked as *j* to denote the slot in the transmission window, $BO_{c}\left(t\right)$ represents the backoff counter for the analyzed slot *t*, and $BO_{s}\left(t\right)$ the backoff stage at slot *t*. These time-discrete stochastic process assuming discrete values conforms a chain that completely characterizes the different transitions of the nodes.

Then, the detailed analytical model is extended in [23], to evaluate the Capture Effect (CE) in a slotted IEEE 802.15.4 mode network. The CE is the ability of wireless devices to recover a packet (or message) when two or more signals of different power overlap with the legitimate packet transmission in the same frequency band. Therefore, the probability that the CE phenomenon occurs will depend on the ratio between the power of the carrier signal ($RSS_{c}$) and the interfering signals ($RSS_{i}$), assuming the overlap of the signals in the transmission time. Therefore, the CE can be evaluated using the signal-to-interference-plus-noise ratio (SINR). In [24], the CE for the unslotted mode is analyzed under the assumption of signals overlap in time. The results of PRR and goodput for different ranges of SINR show that the CE occurs in certain regions for reactive jamming strategy. However, the correlation of the performance metrics with analytical models of the unslotted IEEE 802.15.4 mode is not present. Furthermore, the energy consumption and the constant jamming strategy are not analyzed in [24].

Consequently, to correlate the analytical model previously described with the CE, we made the following modifications and assumptions.

1. The probability $P\{C^{j}\}$ indicates the probability of being in the second sensing phase for all the backoff stages in slotted mode. Conversely, for unslotted mode, this probability accounts for all the sensing phases.
2. The probability that the remaining nodes do not sense the channel in the slot *j* − *D* obtained by $\prod_{i=0}^{NB_{max}}{\left(1-P\left\{S_{i}^{j-D}\right\}\right)}^{\left(N_{c}-N_{i}-1\right)}$ is calculated considering only the second sensing phase. However, for the unslotted mode, this can happen in all sensing phases. Therefore we calculate the probability $P{\left\{Z^{j}\right\}}_{CE}$ for all the (*i*) range.
3. For the joint probability to find the channel free in slot *j* denoted by $f^{j}$, we take into account the same assumption of the probability of the sensing phases for the $P{\left\{Z^{j}\right\}}_{CE}$.

Consequently, we correlate the concept of founding the channel free in the given slot, making $p_{f}^{j}=f^{j}$ to be used in the calculation of the probability $P\{Z^{j}\}$. To compare the different probabilities acquired from the experimental results with the model and the capture effect, the different success probabilities are presented with a fixed value of D=2 for the length of the packets. We also adjust the limit of the axes to the presented in the original works.

### 2.3. Jamming Attacks

To correctly differentiate the impact of the jamming strategies, a parameter that correlates the attacker and the MAC model is mandatory. According to the works the time when the signals collide permits dividing the analysis into different cases [18,25,26]. These cases will be divided according to the field of the frames that are involved in the collision.

The sync bytes and the header field are the most critical fields when the probability of receiving a packet is analyzed. Consequently, the time over the air of the different fields of the packet is essential in the analysis of the attacker. Here, we refer, and we differentiate the analysis of the attacker considering the preamble, sync bytes, and the headers of the packets as reference. Therefore, we determine the synchronization of the interference signal with the first fields of the packet as

(1) $$\phi =\frac{t_{a}-t_{b}}{t_{pr}+t_{sc}+t_{hd}},$$

where $t_{a}$ is the time when the legitimate packet initiates it transmissions, $t_{b}$ the time when the signal interference is generated, and $t_{total}$ the sum of the time that takes to transmit the first fields of the packet denoted as $t_{pr}$ $t_{sc}$ and $t_{hd}$ respectively. Later, the synchronization is transformed from time to slots to analyze each case.

**Figure 2:**
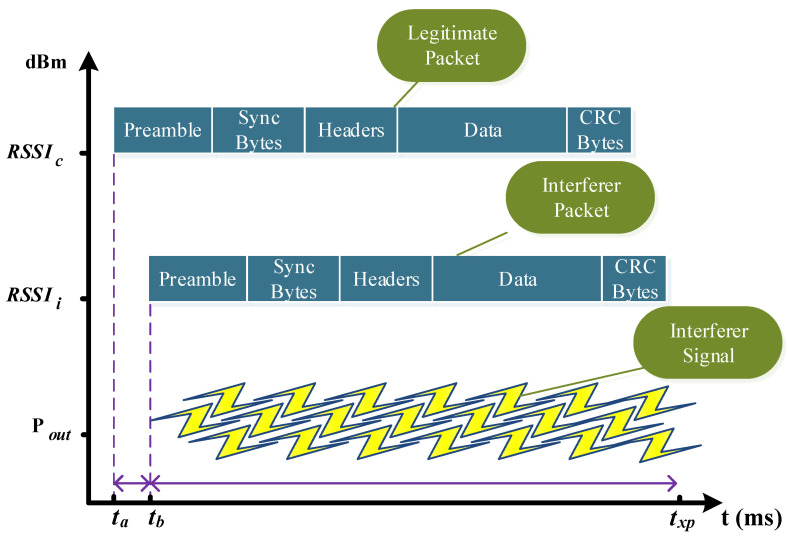
Description of the collision scenario for the different experiments. The difference between $t_{a}$ and $t_{b}$ divides the analysis of the collision and help to understand the behavior of the transmission under jamming attacks. We also show that the jamming strategy can be carried out using a data frame or noise injection.

Depending on the timing and the power of the signals in the communication, collision detection is possible in certain scenarios. This behavior is described as stronger-first and stronger-last scenarios where the legitimate user signal is analyzed with one interference packet on the receiver side, Figure 2 presents a simplification of the realized experiments [25]. Summarizing, when two packets arrive at the receiver, the collision detection will depend on the power of the signals and the time difference between the packets. Therefore, when interference is present in the communications, the analysis is divided into: the users can be identified or the users cannot be identified. This identification process will depend on the range of values that the parameter ϕ can assume. When 0≤ϕ<1 the users cannot be identified, and when ϕ≥1 the users can be identified.

Summarizing, if the preamble, sync, or headers bytes collides with the interferer packet, the receiver cannot synchronize with the legitimate transmitter, or it cannot capture the packet. Therefore, this transmission will be completed loss, and the identification of the users cannot be achieved. We remark that this behavior is conditioned to the power of the signals that need to be determined for each application scenario.

The data modeling of jamming attacks and their impact on the networks is possible considering the parameters of the incidence rate of the signal interference ($r_{j}$), the probability to interfere with the communication ($q_{i}$), the synchronization between the signals (ϕ), and the difference between the power of the interference signal and the power of the legitimate signal (SINR).

The rate of the jammer signal $r_{j}$ will be different for the different strategies and scenarios. The rate will vary depending on the length of the packet, the width of the pulse, the generation of the signal from the activity time in the experiments. Therefore, the different jamming strategies can be analyzed in terms of the used time for transmitting or generate the interference signal divided by the total time of the experiment.

Following the analytical model presented, we have to analyze the probability of the packets collides by the effect of the jamming strategy considering a different number of nodes. For a network composed by *N* nodes, we have that $N_{c}$ contends for the channel in a given slot, and $N_{i}$ interferes nodes are present in the communications. When the interferes nodes are present, they can provoke constructive interference that can be analyzed under the capture effect concept. On the contrary, if the interference nodes do not improve the reception of the packets, it means that destructive interference is present. Therefore, the interferemce node can be interpreted as a jamming device in the network.

Considering $N_{i}=1$ interferes with a destructive interference ϕ<1, the reception of the packet is analyzed using the SINR parameter. Therefore, the complement of the linear regression model that correlates the PRR to the SINR values, gives the probability of jamming

(2) $$\begin{array}{cc} p_{j} & =1-({\mathrm{PRR}-\mathrm{to}-\mathrm{SINR}}_{model}).\\ \; & =1-({\left(1-\frac{1}{2}\exp\left(-\beta_{0}/2\right)\right)}^{8\beta_{1}f}) \end{array}$$

where *f* is the frame size in bytes, $\beta_{0}$ the SINR obtained by experimental measurements, and $\beta_{1}$ corresponds to noise Bandwidth.

Expression (2) is valid only for $N_{i}=1$ despite the numbers of $N_{c}$ nodes present in the network. If more interferences are present in the scenario, then is important to know the relative distance of the interferes to characterize the behavior of the PRR-to-SINR curve, the synchronization ϕ, and the number of interference nodes in the communication channel regard the legitimates nodes. The regression model is accounted for OQPSK modulation in the 2.4 GHz frequency band, 250 Kbps of data rate with NRZ encoding [27].

The constant jamming rate is $r_{j}=1$, given that the interference signal is always present in the communication channel. As a consequence, we could establish that the timing differences between the packets are equal to zero; therefore, full synchronization is achieved.

With ϕ=0, the packets cannot be detected due to the collisions caused by the jammer device. Therefore, the jamming signal is generated independently of the communication process in the network. The jamming probability $p_{s_{jam}}$ can be calculated as

(3) $$p_{s_{jam}}=p_{s}\cdot p_{j}.$$

Therefore, $p_{j}$ will depend on the PRR value for the different ranges of SINR according to the regression model.

The reactive strategy generates interference for some packets or activity in the network. To guarantee the collision, we assume that the jammer knows the time when the transmission is generated in the nodes. The times can be easily decoded by the attackers [28] for different MAC protocols. Therefore, the probability of jamming for the reactive strategy is the same as the constant strategy with different jamming rates that can be configured for different scenarios.

## 3. Experimental Testbed

The evaluation of the performance of networks under jamming attacks has special considerations. Therefore, we propose an experimental testbed to evaluate the performance of the networks using the IEEE 802.15.4 protocol under jamming attacks. To implement the network using the IEEE 802.15.4, we use the XBee S1 devices that work in the 2.4 GHz ISM band with a 250 Kbps data rate. Additionally, it uses the O-QPSK modulation with the DSSS technique for this frequency band and considers the use of 15 channels. The XBee device uses an MC1321x transceiver with an MCU HCS08 family to perform the communications and the communication process described.

The use of a sniffer device is mandatory owing to the implemented network needs to be analyzed in different scenarios. It is important to remark that an XBee device cannot perform this task without influence in some communication processes in the network. For this reason, we choose to implement a sniffer device using a Software Defined Radio (SDR) with the use of GNU Radio software. The SDR corresponds to the NI USRP 2921 that is modified to use a firmware that permits the use of GNU Radio to implement the PHY and MAC layer of the IEEE 802.15.4 protocol [29]. Specifically, we use the base code in [30], and we modified the MAC layer to suppress the communication process. The used codes in the devices can be found in the Appendix A.

**Figure 3:**
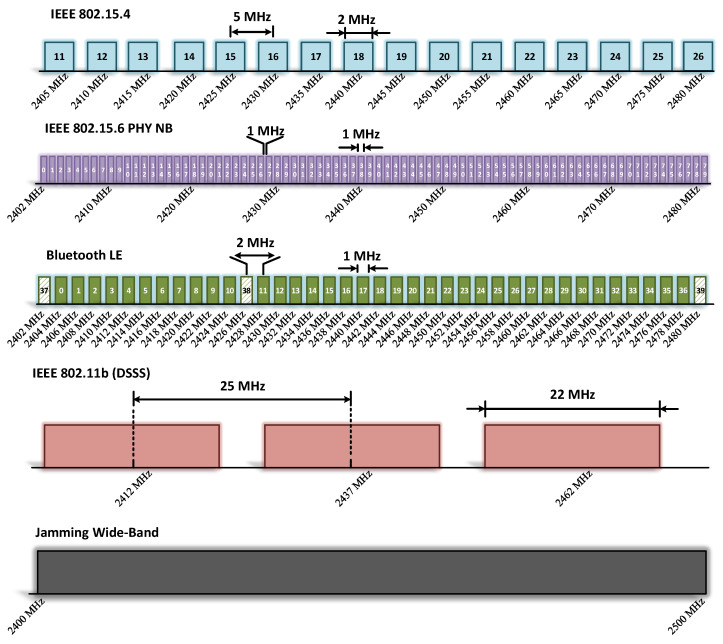
The wideband strategy can impact several channels and communications protocols in the ISM Band. In our work, we focus on analyzing the impact for a network using the IEEE 802.15.4 standard.

The jamming strategies chosen to be analyzed in our work are the reactive and wideband constant jamming strategies. To implement the reactive strategy, we use an XBee device to analyze the impact of that a legitimate node becomes a jammer device under this strategy. For the wideband constant strategy, we use a Voltage Controlled Oscillator (VCO) ZX95-2650+ due to permits the generation of several frequencies. Specifically, we use a frequency generator for the tuning circuit of the VCO to produce a wideband frequency output as shown in Figure 3.

Finally, due to the jamming impacts on all the communication process of the network is important to preserve the synchronization. Specifically, the times of the generation of the legitimate and interference signal are critical. To overcome this problem, we implement a master clock that produces different instructions for the experiments using wires from the master to the slaves. The master clock is used to produce the transmission process in the XBee transmitters and the generation of the interference signal when the reactive strategy is present. The ATMega328P MCU is used for the generation of the master clock and the slave’s clock in the XBee devices. We also upload the code to the MCU that permits the acquisition of relevant data of the communications.

Using this approach, we preserve and control the synchronization of devices despite the presence of jamming attacks. To the best of the authors’ knowledge, this approach in the synchronization to experimentally analyze the effects of jamming attacks in a WSN was not previously proposed and represents one of the core contributions of this work with the inclusion of a sniffer device.

### 3.1. Energy Consumption

To obtain the energy consumption associated with the transmission of the packets and the different processes involved in the communications, we adequate the formula used in [31] to the used transceiver. Moreover, the data are corroborated with experimental measures. To obtain the energy consumption measured in Joules $E_{d}$, we need to know the current consumption for the different modes of each MCU and the operation voltage. Consequently, we can obtain the energy consumption for each device as

(4) $$E_{d}=\left(t_{at}i_{tat}+t_{id}i_{id}+t_{s}i_{s}\right)\;v_{cc}\;,$$

where each *t* is the time in milliseconds that the MCU is in that state with their corresponding current consumption. Specifically, $t_{at}$ is the time of the duty cycle where the device is active, $t_{id}$ is the idle portion of the device in this state, and finally $t_{s}$ are the sleep or low consumption state of the device. Additionally, we use the same time of the length of the experiments $t_{xp}$ to compare the energy consumption in the different scenarios and devices.

#### 3.1.1. XBee

Using the formulas provided by the manufacturer of the XBee devices, we correlate the different times of the communication process with the times present in the formulas of each device [32]. Additionally, using an oscilloscope, the process is analyzed to link with the presented formulas. According to the analyzed pulses in the oscilloscope, we assume that the transmission and the CCA process use almost the same amount of current. On the other hand, the ACK and reception process is similar.

In our experiments, we use 64-bit addressing, 32 μs of byte time and a unicast transmission in a ideal conditions. We also know that the CCA have a fixed value of 128 μs and the ACK 864 μs when is activated. Finally, when the random delay is configured by the use of the backoff exponent, this value is calculated according to the minMacBE and maxMacBE as $t_{Random\;Delay}=2^{\left(BE-1\right)}\times 32$ μs.

#### 3.1.2. ATMega2560

For the ATMega2560, we have the following process running, which makes the transition of the processor in different states.

- The generation of the transmissions and the data packets to be uploaded to the MC1312x MCU.
- The processor continually senses the digital input for the incoming pulse from the master clock to initiate the transmission.
- The reception of the data from the MC1312x MCU and the redirects of the data through the UART port.

Therefore, the ATMega2560 continually changes from the idle to the active states without entering sleep mode. With these processes running in the processor, we can correlate the time active and idle with each of them [33]. As a consequence, the generation of transmission and the reception of the data are the active part $t_{at}=t_{tx}+t_{rx}$. Consequently, the sense of digital input corresponds to idle time $t_{id}=t_{sense}$. Moreover, we consider that the baud rate configured in the code corresponds to 38.4 KBd to estimate the time active for the transmission and reception of the data packets printed to the UART and serial port.

#### 3.1.3. ZX95-2650

The VCO ZX95-2650+ is used to generate a Wide Band Denial jammer with a constant jammer rate [34]. Therefore, the device is always in the experiments, and the power consumption will be constant through the experiment $t_{at}=t_{xp}$. However, we highlight that the energy consumption is calculated considering only the VCO and not the tuning circuit. Finally, Table 1 presents the different current consumption and the respective operation voltage of each device for the calculation of the power.

### 3.2. General Procedure

**Figure 4:**
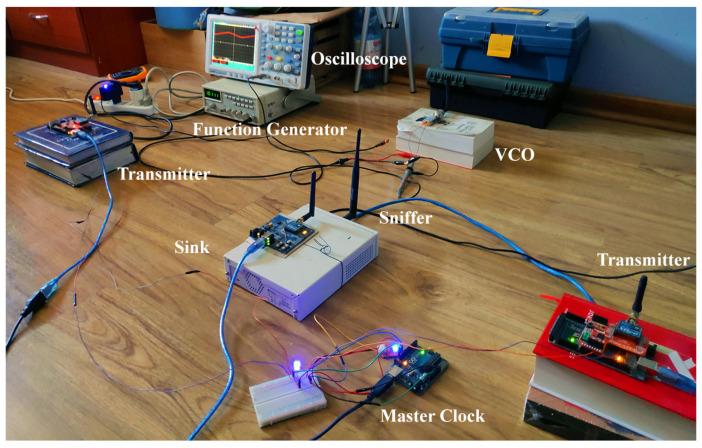
Network devices and instruments are used to obtain experimental data in the indoor environment that consists of a typical bedroom.

The correlation between experimental and analytical results from the system model requires the record of several events. Therefore, each transmitter device on the network is programmed to store the reception, transmissions, and ACK messages in the experiments. The sink node continually records the different received packets for each transmitter and their corresponding RSSI values. We also implement a sniffer to analyze the total behavior of the network in the same position as the sink node. Finally, to correlate the different events recorded by the different devices and analyze the data, we use Wireshark [35] with dedicated code in Python to acquire the performance metrics and transform the RSSI to RSS values. In Figure 4, the different devices and instruments used for the experiments are shown.

The experiments were carried out in an indoor environment that needs to be characterized to validate the acquired data. Therefore, before each experiment, 2000 packets were carried out between one of the transmitters and the sink node to obtain the noise floor and analyze the variance of the RSS values. Therefore, if the RSS values present a higher variance from the ideal scenario, the experiment is discarded. In the ideal scenario, the RSS values are steady across the experiment, without significant variance in the time of the experiment. The average noise floor estimated by experiment data is −82 dBm.

## 4. Measurement and Results Analysis

Several experiments were carried out to analyze the behavior of the implemented WSN using the standard IEEE 802.15.4 with unslotted mode. With the experimental data acquired in a non-jamming scenario and a jamming scenario, we correlate the theoretical model with the experimental data to analyze the impacts of the attacks. Additionally, we characterize and correlate the smart indoor environment channel with the models in the literature. Consequently, the cyberattacks that aim at the physical layer of the WSN are analyzed experimentally and theoretically to improve the existing models.

### 4.1. Channel Characterization

The environment used for the experiment is a typical bedroom with the coexistence of different communication protocols. Therefore, we use the log-normal shadowing path loss model to correlate this analytical model with the experimental data acquired from the RSS values. However, we also analyze the use of Nakagami and Rayleigh models, but we conclude from experimental studies that the chosen model is more accurate for our implemented scenario, which is given as follows:

(5) $$PL\left(d\right)=PL\left(d_{0}\right)+10nlog_{10}\left(\frac{d}{d_{0}}\right)+X_{\sigma }.$$

In our scenario, we obtain that path loss exponent (n) is equal to 1.8 and $PL_{0}=39.62$ dB for a reference distance $d_{0}$ = 1 m and the variable $X_{\sigma }$ is a Gaussian-distributed random variable with zero mean and standard deviation $\sigma_{s}$ that represents the shadowing. These results are consistent with the values presented in the works [36,37,38] for the indoor office environment with LOS component.

**Figure 5:**
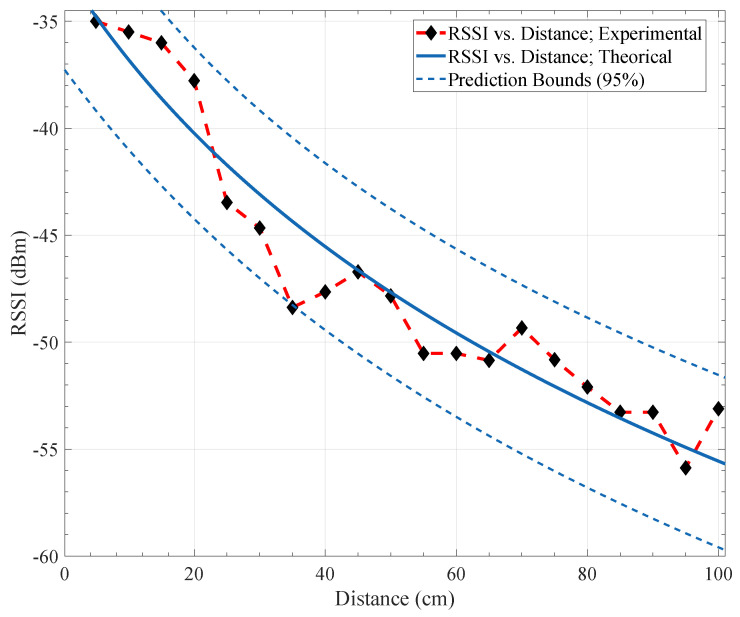
Experimental and theoretical distance dependence of the path loss characterization with *n* = 1.8, σ=1.88, and $X_{\sigma }=53.28$.

In Figure 5 we plot the theoretical and experimental path loss characterization curves for the obtained values of our scenario, as well as prediction bounds of 95%. Using the spectrum analyzer and the transmission of data with one transmitter, we analyzed the RSS values and behavior of the communications on several days and time hours. The results show that the optimal range of hours to perform the experiments is between 8 a.m. and 4 p.m. in the week. In the weekend or outside of this range of hours, the environment has a lot of interference signals, and erratic behavior of the RSS values reported. We also found that channel 12 ($f_{c}$ = 2410 MHz) is the best for communication through the experiments.

The erratic behavior of the RSS values in the used channel for the experiment was also analyzed. As previously stated, the range of hours between 8 a.m. and 4 p.m. in the week is the ideal case that matches with the labor hours. Therefore, the communications protocols using the 2.4 GHz ISM band, such as IEEE 802.11, will be unused owing to the absence of users in their homes. Consequently, this inactivity allows us to perform experiments with little activity on the frequency band. Additionally, it shows us how the coexistence varies the values of the analyzed metrics.

### 4.2. Packet Data Rate and Goodput

**Figure 6:**
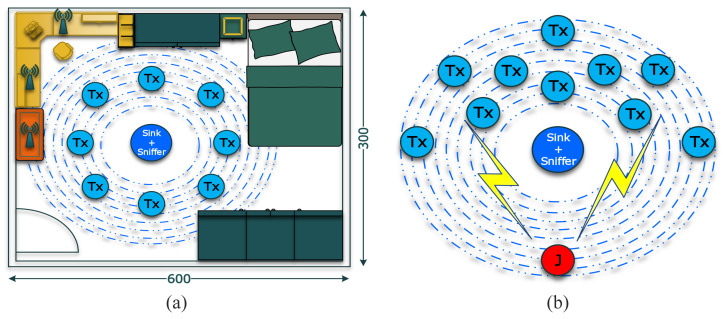
(**a**) The bedroom used for the experiments when the reactive strategy is used, (**b**) The fixed position of the constant jammer for the experiments.

Later, we carried out experiments to analyze the performance of the network composed of the XBee devices under the different jamming strategies. The main difference between the experiments resides in the variation of the BE parameter for the transmitter node to transform into an interferer node. With this modification and the assumptions for our work, this node behaves like a reactive jamming strategy. When the reactive strategy is used, the scenario implemented has a circular distribution of the transmitters nodes to the sink node. Specifically, with the sink node, we deploy the sniffer device in the center of the circle as show in Figure 6a. We also use the same distribution for the constant strategy. However, the distance of the constant jammer device is fixed to one position due to the power output and the impossibility to vary between experiments as displayed in Figure 6b.

The distances between the nodes used in the experiments and the number of nodes suffered from limitations due to the physical space owing to the outbreak of the coronavirus, COVID-19. However, the distances and number of nodes used in this work are in the range of values used in previous works for smart indoor environments [14,17,18,23]). Note that, when the transmitter is used as an interferer, the circular distribution is maintained.

To determine the performance metrics for the scenarios under jamming attacks, we activate the ACK mechanism. Therefore, we have the information of the transmitters, the received packets in the sink, and the registered packets by the sniffer to analyze the behavior. For the first round of experiments, we vary the number of interferers present in the scenario to analyze the performance against one transmitter. Consequently, we start the experiments with one transmitter and one interferer. We remark that the sniffer and the sink node are always implemented in all the experiments.

Using a reactive strategy with one interferer in the scenario, we analyze the PRR from different SINR values. Therefore, we configure a BE=0 for the transmitter and the interferer nodes to generate collisions. The results showed that for values of SINR greater than 4.5 dB, the PRR is almost 0.99. We also corroborate this information by the ACK status reported from the transmitters, which show almost null errors in the transmission.

**Figure 7:**
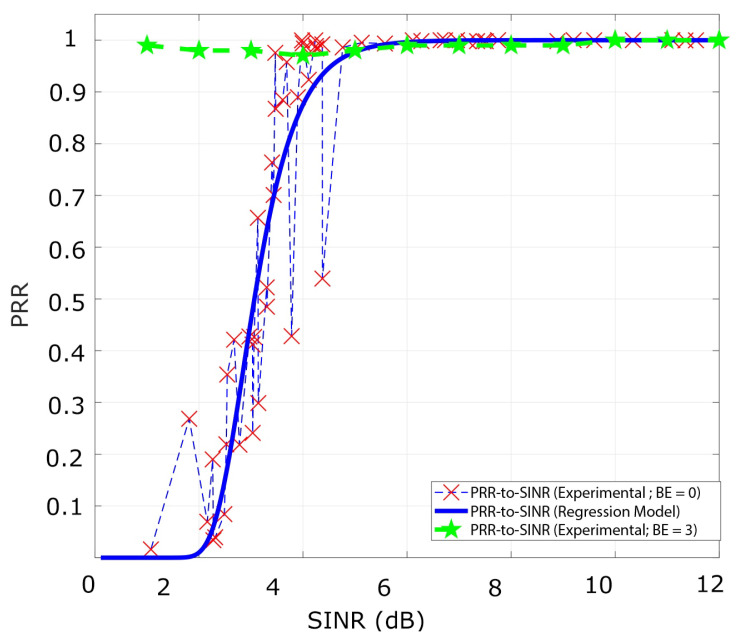
The PRR-to-SINR curve for BE=0 and BE=3 for one transmitter $N_{c}=1$ and one interferer $N_{i}=1$. The curve of the regression model is plotted to show a good agreement with the experimental results obtained.

However, for values of SINR between 0 and 4.5 dB, the legitimate signals overlap with the interference signal that produces different values of PRR, as shown in Figure 7, the PRR-to-SINR values have particular regions of analysis. Additionally, from the curve is deducted that the CE occurs for multiple values of SINR considering one interferer.

**Figure 8:**
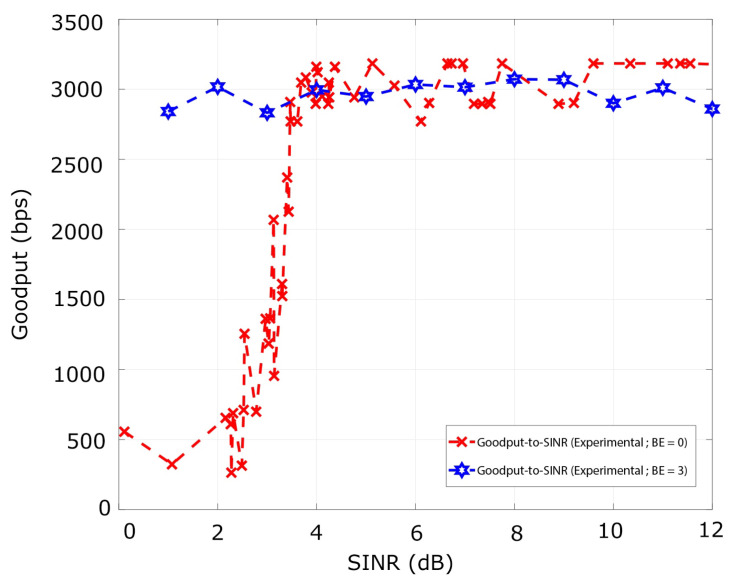
The Goodput-to-SINR curve for BE=0 and BE=3 for one transmitter $N_{c}=1$ and one interferer $N_{i}=1$.

Then we analyze the goodput metric for the same range SINR values with BE=0. The goodput behaves similarly to the curve of PRR in the different regions produced by the SINR range. Specifically, for values of SINR greater than 4.5 dB, both curves converge to a value that for the case of the goodput is 3048.38 bps. Furthermore, for the SINR interval comprehended from [1.07, 4.5] dB, the goodput varies from 556.04 bps to 1006.28 bps. These results are acquired for BE=0, BE=3, and SO=0 and plotted in Figure 8.

Next, using one transmitter, interfering, and the sink node, we analyze the metrics for BE=3. The variation of the window length of the backoff mechanism eliminates the overlap of the signals. Therefore, the curve of PRR-to-SINR is equal to 0.99 for the entire range of values analyzed in the several experiments as presented in Figure 7. Similarly, the goodput converges to a value of 2900 bps for the range between 2.3 to 9 dB. However, for an SINR value equal to 1.8 dB exists a slight decrease of the goodput of 2650 bps owing to the stochastic behavior of the channel across the experiments, as shown in Figure 8.

In summary, for values of SINR higher than 4.5 dB, PRR-to-SINR and goodput-to-SINR curves converge to 0.99 for $N_{i}=1$ and with BE=0 for the reactive strategy.

Then, we increment the number of interferer nodes $N_{i}=2$ to analyze the performance of the transmitter $N_{c}=1$. Following the circular distribution, we deploy the nodes at different distances from the sink node. From here, we only analyze the performance metrics for a total collision scenario, and all the nodes are configured with BE=0. The results show that the interferers generate a constructive interference that completely blocks the reception of packets, as shown in Table 2. Therefore, we focus on finding a threshold value that permits the reception of packets with two interferers.

To encounter the threshold that permits the reception of packets in the sink node for the transmitter node, we use the following methodology. First, we fixed the distance of the transmitter to the sink node. Then, we vary the relative distance of the interferers from the sink and also between them. According to the results acquired, when the second interferer have a threshold of almost 3.1 dBm from the first interferer, the reception of the packets occurs, Table 3 shows some experiments performed that reveals this behavior. Additionally, we remark that the threshold between the interferers and the transmitter follows the assumptions analyzed in the scenario with a $N_{i}=1$.

The reported values of PRR corresponds to the average value of the total devices. The experiments show that for a certain RSS threshold between the interferers, the reception occurs. We also found that a similar threshold between the transmitter and the interferer with the higher RSS value is needed.

Next, we change the interferer node of the reactive strategy to the constant strategy. For this, we deploy the VCO in a fixed position of 60 cm from the sink node. Using the SDR as a spectrum analyzer and correlating the gain of SDR with the XBee devices, we fixed the signal strength in −58 dBm for the jammer. Then, we modify the distance of the XBee transmitter to achieve different SINR values, as shown in Figure 6. The results show that despite the strategy used, the PDR and goodput metrics vary equally. Therefore, the SINR value is critical to ensure the PDR and goodput for the application scenario used in our work.

**Figure 9:**
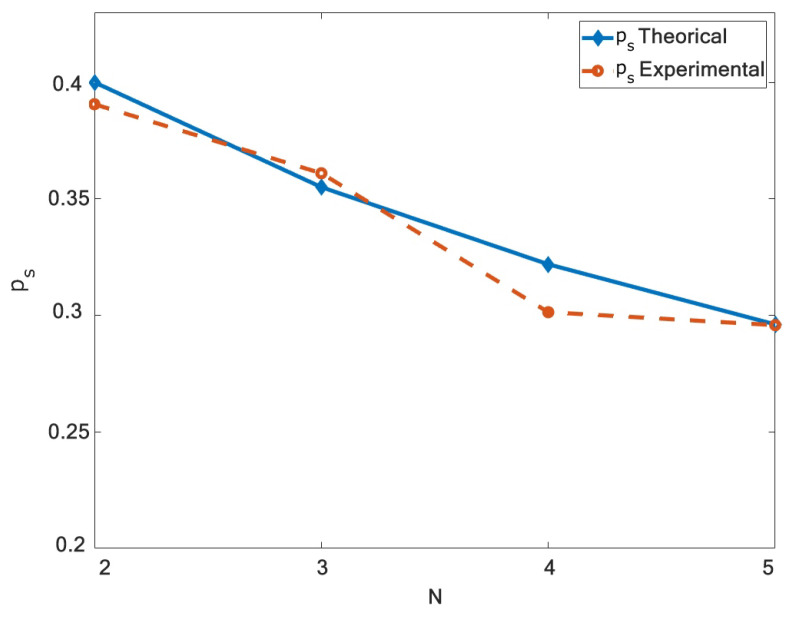
Probability of success $p_{s}$, including the probability of jamming $p_{j}$, is plotted with repect to *N*. A good agreement exists between the experimental and proposed theoretical model.

Finally, we correlate the experimental results of the PDR with the analytical model for the $p_{s}$ performance metric. Using a fixed value of $N_{i}=1$ and $N_{c}=1,2,3,4$, we plot the experimental and theoretical curve of $p_{s}$. For this, we use the protection ratio α=3.1 as used in the work [23] to compare the curves. The experimental curve shows a good agreement with the proposed model under the assumptions used in our work, as shown in Figure 9.

Overall, these results indicate that the impact of both attackers’ strategies is the same for the range of SINR analyzed for the PRR and goodput metrics. Moreover, the acquired experimental data presents a good correlation with the proposed extended model that includes the presence of jamming attacks. Therefore, the WSNs deployed in smart environments under the assumptions of this work could use the PRR and goodput metrics of the extended model to predict the performance of the network under jamming attacks.

### 4.3. RSS and Transmission Status

The RSS and Transmission Status data were acquired in the experiments to analyze their variations in both scenarios. These data are plotted using the timestamp of the packets recorded in the *x*-axis and the RSS value or the status in the *y*-axis. The sniffer is not considered in the following graphics owing to the difference of the hardware and the process to acquire the power of the received signal.

**Figure 10:**
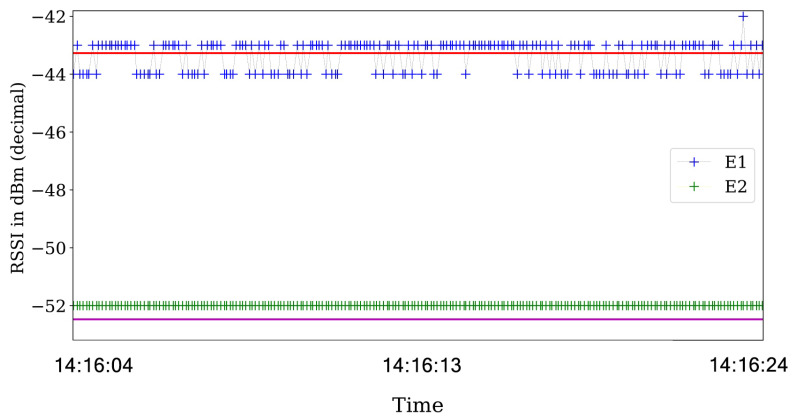
RSS values obtained in an ideal scenario were the red and violet horizontal lines shows the average value across the experiment for each transmitter. The graphs use the timestamp of the packets on the *x*-axis, and are plotted for only 20 s to show the behavior across the experiment.

The first experiments are for the ideal scenario and are performed with the ACKs enable in the communications. For ideal scenarios, the transmission status reported by the ACK is always assessed as a success. Consequently, the RSS values does not present a significant variance as seen in Figure 10.

**Figure 11:**
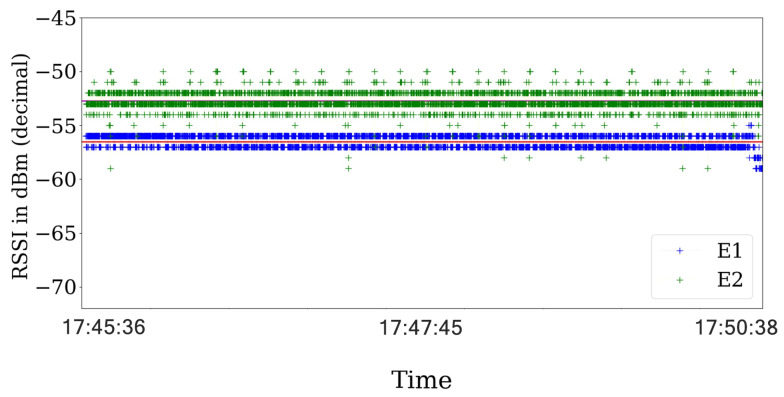
RSS value for the reactive strategy were the red and violet horizontal lines shows the average value across the experiment for each transmitter. The RSS values almost no present notable variations across the experiment.

Then, we analyze the RSS values and their respective ACK status information in scenarios with the jamming strategy active. For the reactive jammer, we plot the RSS values in Figure 11 that show that the RSS values are steady across the experiments.

**Figure 12:**
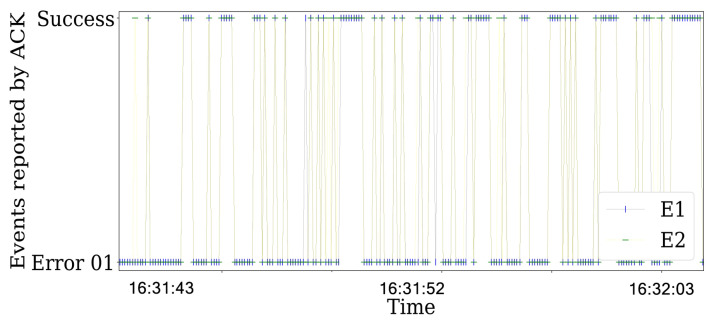
Events reported by the ACK in the communications for SINR=3.1 with BE=0 for the transmitter and the interferer as reactive. The presented behavior is the same for other time periods.

Additionally, we plot the status transmission in Figure 12. In particular, a lot of errors of the 01 type occurs when the jamming strategy is activated. This means that the packet that was transmitted was reported as a failure transmission due to collisions.

**Figure 13:**
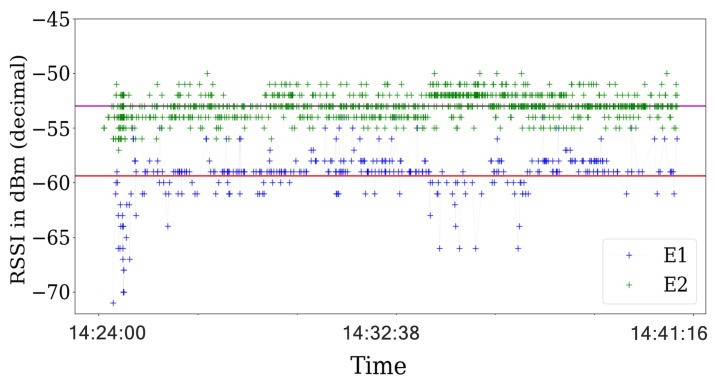
RSS value for the constant strategy were the red and violet horizontal lines shows the average value across the experiment for each transmitter. The RSS values presents a higher variance than the reactive strategy.

Finally, using the same configuration of the experiments with the reactive jammer, we implement the constant jamming strategy. In the same way as the reactive jammer status report, the constant jammer presents a lot of errors. However, the major difference is in the variance of the RSS values as shown in Figure 11 for the reactive strategy and in Figure 13 for the constant strategy.

We also analyze the variance and standard deviation of the RSS values for different values of SINR and jamming strategies. For the reactive strategy, there exists a correlation between the variance and the SINR for the round of experiment realized. When the SINR is lower than 4 dB, the variance present higher values for the range. However, for values of SINR above 7 dB, the variance achieves its lowest value of 0.3 as shown in Table 4.

Nevertheless, the constant jamming behavior is different for all the range of values of SINR analyzed. The variance presents values above of 1.22 for all of the SINR values obtained in the different experiments. Contrary to the reactive strategy, the constant strategy impacts the RSS values reported by the sensing states. As a consequence, the RSS can be used to improve the detection of attacks in the communication channel, under the assumptions of this work.

**Figure 14:**
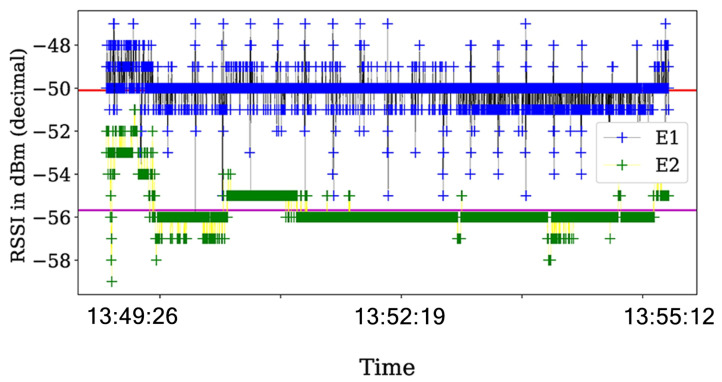
The ACK reports across the experiments and the presence of the impulsive noise in the range of 13:48:57 to 13:49:17 for both transmitters. The status reports expose that for that range of timestamps, the ACK reports a failure in the transmission process. However, the presence of interference for the E1 transmitter across the experiments does not lead to error in the transmission.

The channel also suffered from other sources of interference as impulsive noises. This phenomenon produces a high variance of RSS values in certain time ranges and triggers an error in the status of the transmission reported by the ACK. In Figure 14 the impulsive burst noise appears for the timestamp values between 13:48:57 to 13:49:17. This effect generates the highest RSS values of the experiment from the average values acquired in the experiment.

**Figure 15:**
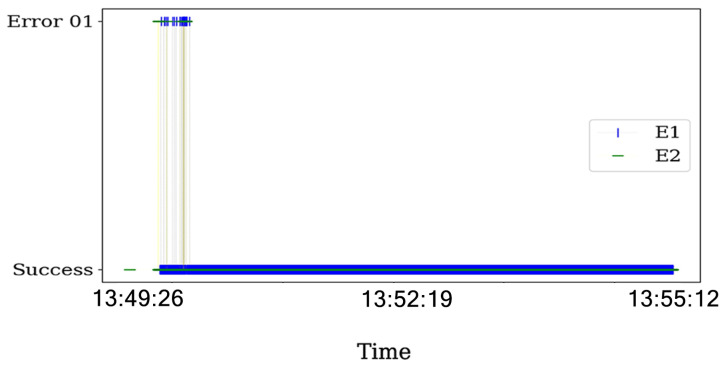
RSS values across the experiments, and the presence of impulsive noise in the time range of 13:48:57 to 13:49:17 for both transmitters.

As a consequence, the RSS has the highest values across the experiment for this timestamp range for both transmitters. Summarizing, this behavior shows that the CE can cope with the presence of impulsive noise (or interference) for some packets. For the packets that are not captured, the ACK mechanism can detect the collision and report the transmission failure as show in Figure 15. We emphasize that the transmitter E1 is affected by interference during the entire experiments, but does not lead to errors in the transmission. According to the study of the interferences done in Section 2, the collision occurred outside of the preamble or header bytes of the packet.

The variance that the noise of the burst type and the constant strategy provokes in the RSS values are critical for localization applications [39]. To correctly distinguish the presence of attackers, the analysis of various performance metrics as RSS, PDR, and goodput is mandatory. Therefore, it is important to use countermeasures that combine use various performance metrics with the RSS values to analyze the presence of attackers.

### 4.4. Energy Consumption

The energy consumption is calculated using the assumption and associations described in Section 3.1 for the single packet transmission. Consequently, we need to calculate the energy consumption for the total packets that are transmitted in the time of the experiment $t_{xp}$ in each scenario. Finally, we correlate the ideal and worst-case scenario with the XBee processes to obtain the time in each scenario for the ATMega2560. Using the time of the experiment and the total transmissions generated by the master clock, we know that a 9000 process of transmission will be generated. Hence, in Table 5, we present the different timing and energy consumption for the transmitter in each scenario.

In the case of the energy consumption for the jammer devices, we have to calculate separately. For the reactive strategy, the jammer device corresponds to the XBee device used for the legitimate transmitter. Therefore, the electrical characteristics are the same for the calculations. However, the transmission and reception process differs dramatically. As explained earlier, we assume that the reactive jammer has complete knowledge as discussed in [28]. Therefore, we emulate the reactive strategy transmitting at the same time instant of the legitimate transmitters with a constant scanning of activity in the communication channel. Consequently, the device only presents $t_{at}$ time that corresponds to the transmission process of packets and is equal to $t_{xp}$. The same assumption is for the constant jammer with their respective electrical characteristics.

Having stated how to calculate the energy consumption for each device, we contrast the results. For the transmitter, we sum the energy consumption of XBee and ATMega2560 devices in the jammer scenario. Then, we calculate the energy consumption of each jammer device with the previously stated times and with the electrical characteristics presented in Table 1. The results of total energy consumption for the different devices are presented in Table 6.

The results are consistent with the methodology and the works that analyze these types of strategies. The constant jammer has higher energy consumption than the other devices and the reactive strategy. In particular, the energy consumption of constant strategy is 40% higher than the transmitter and 118% from the reactive jammer.

**Figure 16:**
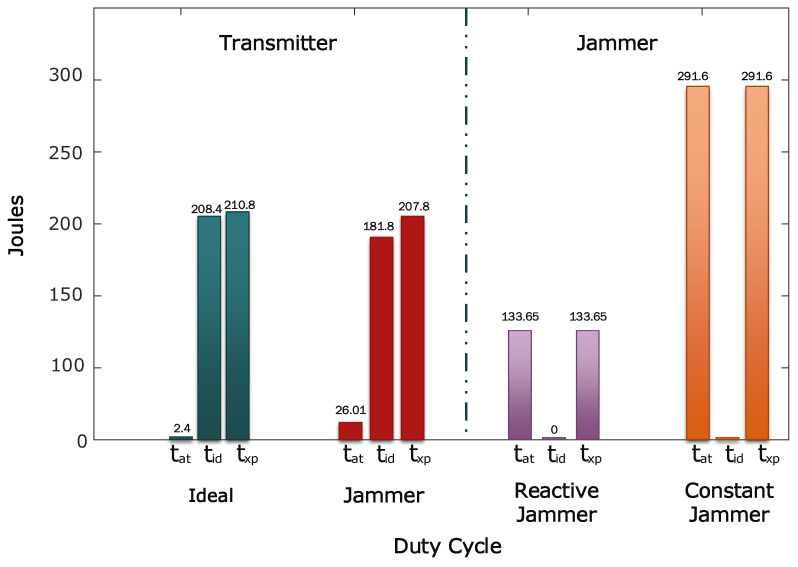
Bar plot of the energy consumption for the transmitter in the ideal and jammer scenario for the different states of the duty cycle. Additionally, the energy consumption of both jammers is presented.

However, is interesting to remark that the energy consumption of the transceiver does not present great variations. Moreover, in the worst-case scenario, the XBee has a lower energy consumption than the ideal scenario with 207.82 J from 210.80 J. This behavior is because the current consumption in the transmitting mode is lower than the idle mode. As a consequence, when a lot of retransmission occurs, the transmitting mode generates more active time, and this compensates for the energy consumption of the ideal scenario as shown in Figure 16. A similar behavior accounts for the reactive jamming implemented in the XBee devices.

Additionally, the energy consumption of the transmitters in the worst scenario from the reactive strategy is 35% lower with a incidence rate of $r_{j}=\;0.19$ that corresponds to the ratio between the used time to generate the interference 173 s from the total time of the experiment $t_{xp}$. As a consequence, the attacker could severely impact the lifetime of the networks using transceivers with similar characteristics. We recommend the use of devices with higher energy efficiency between the transmitting and idle state to improve the lifetime of the network and the implementation of sleep times is mandatory to extend the lifetime. Additionally, the detection of the attacker is mandatory to preserve the integrity of the users in smart environments.

For applications with strict QoS requirements, reactive jammers can expose users to several risks owing to that the jammer has a better energy efficiency than the nodes, which severely compromises the lifetime of the devices. For a 35% lower energy consumption, a reactive strategy can fully discharge the battery of smart devices. Therefore, we recommend that detection algorithms should use RSS values, PDR metric, and the ACK frames status report to improve the integrity of the devices and the network. A reasonable approach to tackle this issue could be to use AI or machine learning techniques to improve the detection of these types of attacks.

## 5. Conclusions

In this study, we proposed an experimental testbed to evaluate the IEEE 802.15.4 networks based on WBANs using the CSMA/CA unslotted mode under jamming attacks. The performance measures obtained by the experimental testbed were correlated with analytical models. Results showed a good correlation of the performance metrics for the ideal and the attacker scenarios. In particular, we acquired experimental data that shows that for one interferer in the communication, the SINR allows us to predict the behavior of the PRR using the regression model. For our experimental scenario, considering one interferer and a maximum of 4 contending nodes, an SINR of 3.1 dB ensures the reception of half of the total packets. Additionally, for values of SINR greater than 4.5 dB, the goodput and PRR converge and have the same behavior as the ideal scenario without the presence of attackers. Conversely, when two interferers are present in the network, the range of SINR and the PRR-to-SINR regression model varies drastically. However, we found that with a threshold of 3.1 dBm between the interferers nodes, the reception of packets is possible. Additionally, a similar threshold value between the transmitter and the interferer with higher a RSS from the sink is needed to guarantee the reception.

As a consequence, we extended the analytical model and incorporated the jamming probability. Using the PRR-to-SINR regression model, we found the jamming probability for the evaluated scenario. The results show a good agreement between the extended analytical model and the experimental data acquired. The modeling approach is valid under the assumptions used in this work and can be extended to a major number of contending nodes or using other jamming strategies. However, if more interferer nodes are present, the regression model needs to be adjusted to the evaluated scenario.

Further experimental works are needed to estimate the performance of the network for two or more interferers with the analyzed performance metrics. Additionally, with the experimental data, the PRR-to-SINR regression model can be adjusted to include the presence of more interferers. With these analyses, the generation of countermeasures in WSNs deployed in smart environments will be improved against jamming attacks.

## Figures and Tables

**Table 1:** Current consumption for each state and operation voltage for each device used in the experimental testbed [29,32,33]. The consumption of the devices used for the synchronization and the tuning circuit are not considered for the comparison analysis.

Device	Current Consumption			Voltage
	Transmission	Idle	Sleep	
XBee	45 mA	50 mA	10 μA	3.3 V
ATMega2560	4 mA	13 mA	15 μA	5 V
ZX95-2560+	27 mA	-	-	12 V

**Table 2:** PRR values for $N_{i}=2$ and $N_{c}=1$, using BE=0. The PRR is almost zero independent of the SINR values. Two experiments are shown to graph the behaviour.

Transmitter	RSS (dBm)	SINR (dB)	ACK		Packets Capture (Sniffer + Sink)	PRR (×10${}^{-4}$)
			Success	Error		
t1	−58.66	8.37	0	2955	23	6.70
i1	−66.51		0	2925	3	10.30
i2	−67.50		0	2910	1	34.00
t1	−56.01	3.10	0	2856	2	17.50
i1	−56.51		0	2920	2	6.90
i2	−59.33		0	2910	3	10.30

**Table 3:** Several experiments were performed to acquire the threshold in RSS from the sink node between the devices.

Transmitter	RSS (dBm)	SINR (dB)	PRR
t1	−46.14	2.48	0.25
i1	−50.10		
i2	−54.11		
t1	−45.92	2.63	0.26
i1	−50.11		
i2	−54.12		
t1	−47.77	6.16	0.29
i1	−51.20		
i2	−54.31		
t1	−47.81	7.82	0.33
i1	−50.98		
i2	−54.10		

**Table 4:** Variance and standard deviation for different experiments and for reactive and constant jamming strategies. The variance of the experiments with a reactive jammer is lower than the constant strategy despite the SINR value.

Jamming Strategy	SINR (dB)	$\sigma^{2}$	s
Reactive	1.08	0.96	0.92
	2.78	0.39	0.58
	4.02	0.53	0.68
	7.75	0.30	0.55
Constant	1.18	1.20	1.45
	2.05	2.54	6.44
	4.49	8.49	2.91
	7.10	0.87	0.76

**Table 5:** The duty cycle and the energy consumption in Joules is calculated for each device that generates the transmission of the packets for the scenarios in the presence and in absence of the attackers.

Device	Scenario	Duty Cycle		Joules
		*t_at_*	*t_id_*	
XBee	Ideal	15 s	885 s	148.25
	Jammer	173 s	727 s	145.66
ATMega2560	Ideal	8.68 s	891.32 s	62.57
	Jammer	16.45 s	883.55 s	62.18

**Table 6:** Energy consumption for each device and role in the experiments are tabulated. The transmitter only considers the jammer scenario to compare the results.

Device/Rol	Duty Cycle		Joules
	*t_at_*	*t_id_*	
Transmitter	173 s	727s	207.82
Reactive Jammer	900 s	0 s	133.65
Constant Jammer	900 s	0 s	291.60

## Data Availability

Not applicable.

## Abbreviations

The following abbreviations are used in this manuscript:

ASK	Amplitude Shift Keying
BE	Backoff Exponent
BER	Bit Error Rate
CA	Collision Avoidance
CE	Capture Effect
CCA	Clear Channel Assesment
CSMA	Carrier-sense multiple access
CTS	Clear To Send
DSSS	Direct-Sequence Spread Spectrum
DoS	Denial of Service
DTMC	Discrete Time Markov Chain
ED	Energy Detection
FFD	Full-Function Device
IoT	Internet of Things
IPv6	Internet Protocol Version Six
ISM	Industrial Scientific and Medical
LLN	Low Power and Lossy Networks
LQI	Link Quality Indicator
MAC	Medium Access Control
QoS	Quality of Service
O-QPSK	Offest-Quadrature Phase Shift Keying
P2P	Peer-to-Peer
PCA	Priorized Contention Access
PDR	Packet Data Rate
PHY	Physical
PSK	Phase Shift Keying
PPDU	Presentation Protocol Packet Data Unit
RFD	Reduced-Function Device
RPL	Routing Protocol for Low power and Lossy Networks
RSS	Received Signal Strength
RSSI	Received Signal Strength Indicator
RTS	Request To Send
SDR	Software Defined Radio
SINR	Signal Interference-plus-Noise Ratio
SNR	Signal to Noise Ratio
TDMA	Time Division Multiple Access
TSCH	Time Slotted Channel Hopping
WLAN	Wireless Local Area Network
WSN	Wireless Sensor Network

## Symbols

The following symbols are used in this manuscript:

$\beta_{0}$	SINR obtained by experimental measurements
$\beta_{1}$	Noise Bandwidth
ϕ	Synchronization between legitimate signal and interfering signal
*D*	Length of the packet in slots used
$E_{d}$	Energy consumption of the device
*f*	frame size in bytes
$f^{j}$	Joint probability to find the channel free in slot *j*
*i*	Backoff Stage
*j*	Slot of time expressed in backoff units
*N*	Number of nodes in the network
$N_{c}$	Number of contending nodes
$N_{i}$	Number of interfering nodes
$p_{j}$	Probability that a packet in the transmission collides with the jamming signal
$p_{s}$	Probability that a generic packet is successfully transmitted
$p_{s_{j}am}$	Probability that a generic packet is successfully transmitted with the presence of jamming
$P\{C^{j}\}$	Probability of being in the second sensing phase
PL(d)	The distance dependence of the pathloss in dBm
$P\{S_{i}^{j-D}\}$	Probability of being in the *i* sensing phase for the j−D slot
$P\{Z^{j}\}$	Probability that a successful transmission ends in slot *j*
$q_{i}$	Probability of the jamming attack to interfere with the communication
$r_{j}$	Rate of the signal interference from the total time of transmission of the legitimate signal
$t_{a}$	Time when the legitimate packet initiates it transmission
$t_{b}$	Time when the signal interference is generated
$t_{s}$	Sleep time in milliseconds
$t_{at}$	Active time in milliseconds
$t_{hd}$	Time of transmission of the headers fields of the packet
$t_{id}$	Idle time in milliseconds
$t_{pr}$	Time of transmission of the preamble fields of the packet
$t_{rx}$	Time to receive a generic packet
$t_{sc}$	Time of transmission of the synchronization fields
$t_{tx}$	Time to transmit a generic packet
$t_{xp}$	Total time of the experiment in milliseconds
$t_{sense}$	Time of the sensing phase in CCA algorithm

## Appendix A

The codes used in this work can be found in https://github.com/xb33/Thesis (accessed on 10 June 2021).

## References

[1] Andrade R.O., Yoo S.G., Tello-Oquendo L., Ortiz-Garcés I. (2020). A Comprehensive Study of the IoT Cybersecurity in Smart Cities. IEEE Access.

[2] Meneghello F., Calore M., Zucchetto D., Polese M., Zanella A. (2019). IoT: Internet of Threats? A survey of practical security vulnerabilities in real IoT devices. IEEE Internet Things J..

[3] Yang Y., Wu L., Yin G., Li L., Zhao H. (2016). A Survey on Security and Privacy Issues in Internet-of-Things. IEEE Internet Things J..

[4] Amin Y.M., Abdel-Hamid A.T. (2016). A Comprehensive Taxonomy and Analysis of IEEE 802.15.4 Attacks. J. Electr. Comput. Eng..

[5] Zou Y., Zhu J., Wang X., Hanzo L. (2016). A Survey on Wireless Security: Technical Challenges, Recent Advances, and Future Trends. Proc. IEEE.

[6] Vadlamani S., Eksioglu B., Medal H., Nandi A. (2016). Jamming attacks on wireless networks: A taxonomic survey. Int. J. Prod. Econ..

[7] Sadeghi A.R., Wachsmann C., Waidner M. (2015). Security and Privacy Challenges in Industrial Internet of Things. Proceedings of the 52nd Annual Design Automation Conference (DAC’15).

[8] Mullet V., Sondi P., Ramat E. (2021). A Review of Cybersecurity Guidelines for Manufacturing Factories in Industry 4.0. IEEE Access.

[9] Gupta M., Abdelsalam M., Khorsandroo S., Mittal S. (2020). Security and Privacy in Smart Farming: Challenges and Opportunities. IEEE Access.

[10] Cupp O.S., Walker D., Hillison J. (2004). Agroterrorism in the U.S.: Key Security Challenge for the 21st Century. Biosecur. Bioterror. Biodef. Strategy Pract. Sci..

[11] Hassija V., Chamola V., Saxena V., Jain D., Goyal P., Sikdar B. (2019). A Survey on IoT Security: Application Areas, Security Threats, and Solution Architectures. IEEE Access.

[12] Islam S.M.R., Kwak D., Kabir M.H., Hossain M., Kwak K.-S. (2015). The Internet of Things for Health Care: A comprehensive survey. IEEE Access.

[13] Usman M., Ashgar M.R., Ansari I.S., Qaraqe M. (2018). Security in Wireless Body Area Networks: From In-Body to Off-Body Communications. IEEE Access.

[14] Ashraf F., Hu Y., Kravets R.H. Bankrupting the jammer in WSN. Proceedings of the 2012 IEEE 9th International Conference on Mobile Ad-Hoc and Sensor Systems (MASS 2012).

[15] Wood A.D., Stankovic J.A., Zhou G. DEEJAM: Defeating Energy-Efficient Jamming in IEEE 802.15.4-based Wireless Networks. Proceedings of the 2007 4th Annual IEEE Communications Society Conference on Sensor, Mesh and Ad Hoc Communications and Networks.

[16] Strasser M., Danev B., Čapkun S. (2010). Detection of Reactive Jamming in Sensor Networks. ACM Trans. Sen. Netw..

[17] Xu W., Trappe W., Zhang Y., Wood T. (2005). The Feasibility of Launching and Detecting Jamming Attacks in Wireless Networks. Proceedings of the 6th ACM International Symposium on Mobile Ad Hoc Networking and Computing (MobiHoc’05).

[18] Wilhelm M., Martinovic I., Schmitt J.B., Lenders V. (2011). Short paper: Reactive jamming in wireless networks: How realistic is the threat?. Proceedings of the Fourth ACM Conference on Wireless Network Security (WiSec’11).

[19] Buratti C., Verdone R. (2009). Performance Analysis of IEEE 802.15.4 Non Beacon-Enabled Mode. IEEE Trans. Veh. Technol..

[20] Rashid B., Rehmani M.H. (2016). Applications of wireless sensor networks for urban areas: A survey. J. Netw. Comput. Appl..

[21] IEEE Std 802.15.4-2015 (2015). IEEE Standard for Low-Rate Wireless Networks.

[22] Buratti C. (2010). Performance Analysis of IEEE 802.15.4 Beacon-Enabled Mode. IEEE Trans. Veh. Technol..

[23] Gezer C., Buratti C., Verdone R. Capture Effect and in IEEE and 802.15.4 Networks and Modelling and Experimentation. Proceedings of the 2010 5th International Symposium on Wireless Pervasive Computing (ISWPC).

[24] Lopez N.A., Azurdia-Meza C.A., Montejo-Sanchez S., Valencia C. Experimental Evaluation of Capture Effect in an IEEE 802.15.4 WSN based on Unslotted-CSMA/CA. Proceedings of the IEEE Latin-American Conference on Communications (LATINCOM 2020).

[25] Whitehouse K., Woo A., Jiang F., Polastre J., Culler D. Exploiting the capture effect for collision detection and recovery. Proceedings of the Second IEEE Workshop on Embedded Networked Sensors (EmNetS-II).

[26] Yuan D., Hollick M. Let’s talk together: Understanding concurrent transmission in wireless sensor networks. Proceedings of the 38th Annual IEEE Conference on Local Computer Networks Workshops.

[27] Zuniga M., Krishnamachari B. Analyzing the Transitional Region in Low PowerWireless Links. Proceedings of the 2004 First Annual IEEE Communications Society Conference on Sensor and Ad Hoc Communications and Networks.

[28] Cheng X., Shi J., Sha M. (2019). Cracking the Channel Hopping Sequences in IEEE 802.15.4e-Based Industrial TSCH Networks. Proceedings of the International Conference on Internet of Things Design and Implementation (IoTDI’19).

[29] National Instruments Specifications USRP-2921; Software Defined Radio Device. https://www.ni.com/documentation/en/usrp-software-defined-radio-device/latest/specs-usrp-2921/specs/.

[30] Bloessl B., Leitner C., Dressler F., Sommer C. A GNU Radio-Based IEEE 802.15.4 Testbed. Proceedings of the 12th GI/ITG KuVS Fachgespräch Drahtlose Sensornetze (FGSN 2013).

[31] Lopez N., Azurdia-Meza C., Valencia C., Montejo-Sanchez S. On the performance of 6LoWPAN using TSCH/Orchestra mode against a jamming attack. Proceedings of the IEEE Chilean Conference on Electrical, Electronics Engineering, and Informatics and Communication Technologies.

[32] Digi International Inc XBee/XBee-PRO S1 802.15.4 (Legacy) User Guide. https://www.digi.com/resources/documentation/digidocs/pdfs/90000982.pdf.

[33] Freescale Semiconductor MC13211/212/213, ZigBee Compliant Platform 2.4 GHz Low Power Transceiver for the IEEE 802.15.4 Standard plus Microcontroller. Technical Report. https://www.nxp.com/docs/en/data-sheet/MC1321x.pdf.

[34] Mini-Circuits Voltage Controlled Oscillator ZX95-2650+.

[35] Combs G. (2021). Wireshark. https://www.wireshark.org/.

[36] Molisch A.F., Balakrishnan K., Cassioli D., Chong C.C., Emami S., Fort A., Karedal J., Kunisch J., Schantz H., Schuster U. (2004). IEEE 802.15.4a Channel Model and Final Report.

[37] Takizawa K., Aoyagi T., Takada J.I., Katayama N. Channel Models for Wireless Body Area Networks. Proceedings of the 30th Annual International IEEE EMBS Conference.

[38] Francisco R.D. Indoor Channel Measurements and Models at 2.4 GHz in a Hospital. Proceedings of the 2010 IEEE Global Telecommunications Conference GLOBECOM 2010.

[39] Bianchi V., Ciampolini P., Munari I.D. (2019). RSSI-Based Indoor Localization and Identification for ZigBee Wireless Sensor Networks in Smart Homes. IEEE Trans. Instrum. Meas..

